# Prognostic Value of the Lactate-to-Albumin and C-Reactive Protein-to-Albumin Ratios in COVID-19–Associated ARDS

**DOI:** 10.3390/jcm15135294

**Published:** 2026-07-07

**Authors:** Sultan Almuntashiri, Yazeed Adel M. Alsubhi, Ziyad Khalid B. Alhelali, Abdulrahman Fraih M. Alanazi, Meshari Mohammed B. Alamri, Bader M. Alshoumr, Saleh Alghamdi, Mohammad Hajaj Said Almermesh, Talib Hussain, Sirajudheen Anwar

**Affiliations:** 1 Department of Clinical Pharmacy, College of Pharmacy, University of Ha’il, Ha’il 55473, Saudi Arabia; s.almuntashiri@uoh.edu.sa (S.A.);; 2 Department of Health Informatics, College of Public Health and Health Informatics, University of Ha’il, Ha’il 55473, Saudi Arabia; 3 Medical and Diagnosis Research Center, University of Ha’il, Ha’il 55473, Saudi Arabia; 4 Department of Clinical Pharmacy, Faculty of Pharmacy, Al-Baha University, Al-Baha 65779, Saudi Arabia; 5 Department of Pharmacology and Toxicology, College of Pharmacy, University of Ha’il, Ha’il 55473, Saudi Arabia

**Keywords:** COVID-19–associated ARDS, lactate-to-albumin ratio (LAR), C-reactive protein-to-albumin ratio (CAR), 30-day mortality, prognostic biomarkers

## Abstract

**Background**: Coronavirus disease 2019 (COVID-19) is frequently complicated by acute respiratory distress syndrome (ARDS), which is associated with high mortality. The lactate-to-albumin ratio (LAR) and C-reactive protein-to-albumin ratio (CAR) have been proposed as prognostic markers in critical illness, but their comparative performance in COVID-19–associated ARDS is not well established. **Methods**: We conducted a retrospective cohort study using the MIMIC-IV database, which contains data from Beth Israel Deaconess Medical Center, United States, including COVID-19 ICU admissions from 2020 to 2022. Adult COVID-19 patients with available PaO_2_/FiO_2_, lactate, albumin, and C-reactive protein measurements within the first 48 h of ICU admission were included. Receiver operating characteristic analysis, Kaplan–Meier survival analysis, and Cox proportional hazards regression were performed. **Results:** Of the 3620 patients screened, 66 met the inclusion criteria; 36 survived and 30 died. Non-survivors had significantly higher LAR and CAR values at ICU admission. In ROC analyses, LAR demonstrated moderate discrimination for 30-day mortality (AUC 0.706) and showed higher discriminatory performance than CAR in most subgroup analyses. Among ARDS patients, LAR showed moderate predictive ability (AUC 0.693), compared with CAR (AUC 0.655). In severe ARDS, both markers demonstrated improved discrimination (0.722 AUC for LAR and 0.797 for CAR). High LAR was associated with significantly higher 30-day mortality in all ARDS patients (60.5% vs. 24.0%; *p* = 0.0056) and severe ARDS patients (72.0% vs. 20.0%; *p* = 0.0022). Elevated CAR was also associated with increased mortality, particularly in severe ARDS (73.1% vs. 14.3%; *p* < 0.001). In multivariable Cox regression, LAR remained independently associated with 30-day mortality. **Conclusions**: LAR demonstrated independent prognostic value for 30-day mortality in critically ill COVID-19 patients with ARDS. CAR showed variable performance. These readily available biomarkers, particularly LAR, may aid early risk stratification although larger studies are needed to confirm these findings.

## 1. Introduction

Coronavirus disease 2019 (COVID-19) has been associated with a wide spectrum of clinical severity, ranging from mild respiratory symptoms to critical illness and death [1]. COVID-19 has been linked to substantial morbidity and mortality, particularly in those requiring intensive care unit (ICU) admission [2]. Severe diseases are frequently accompanied by respiratory failure, hemodynamic instability, and multi-organ dysfunction, all of which contribute to poor clinical outcomes [1]. Notably, a significant proportion of critically ill patients with COVID-19 develop acute respiratory distress syndrome (ARDS), which remains one of the leading causes of ICU admission and mortality [3]. Recent systematic reviews meta-analyses reported that COVID-19–associated ARDS occurs in approximately one-third of patients with severe disease, with mortality approaching 40% among affected patients [4,5].

ARDS is a severe form of acute lung injury characterized by diffuse alveolar damage, increased pulmonary vascular permeability, and non-cardiogenic pulmonary edema [6]. These processes lead to alveolar fluid accumulation, surfactant dysfunction, and impaired gas exchange across the alveolar–capillary barrier, resulting in refractory hypoxemia [7]. ARDS is clinically stratified into mild, moderate, and severe categories based on the partial pressure of arterial oxygen to fraction of inspired oxygen (PaO_2_/FiO_2_) ratio according to the Berlin definition, with severe ARDS representing the most advanced stage of disease and the highest risk of mortality [8,9]. In this setting, profound hypoxemia, extensive inflammatory lung injury, and systemic organ stress contribute to poor outcomes despite supportive intensive care management [10].

Severe COVID-19–associated ARDS is characterized not only by extensive pulmonary injury but also by systemic inflammation, endothelial dysfunction, tissue hypoxia, and metabolic abnormalities [7,11,12]. These processes contribute to disease progression and poor clinical outcomes and have led to increasing interest in biomarkers that reflect inflammatory burden and metabolic stress. Such biomarkers may provide valuable prognostic information and support early risk stratification in patients with COVID-19–associated ARDS.

Inflammation- and metabolism-based biomarkers have been increasingly explored for early risk stratification in critically ill patients. Among these biomarkers, lactate, C-reactive protein (CRP), and albumin have received considerable attention because they reflect distinct but complementary aspects of critical illness. Lactate may reflect tissue hypoxia, impaired perfusion, and metabolic stress [13], whereas C-reactive protein (CRP) reflects systemic inflammatory burden [14]. Albumin is a negative acute-phase reactant and may reflect inflammation, capillary leak, nutritional status, and overall physiological reserve [15]. In patients with COVID-19 and COVID-19–associated ARDS, elevated lactate and CRP levels as well as hypoalbuminemia have been associated with greater disease severity and worse clinical outcomes [16,17,18]. Because lactate, CRP, and albumin are routinely measured in critically ill patients, they may provide readily available prognostic information without requiring additional testing.

To integrate information from these complementary biomarkers, composite indices such as the lactate-to-albumin ratio (LAR) and C-reactive protein-to-albumin ratio (CAR) have been increasingly investigated as prognostic markers in critical illness. LAR has been shown to be associated with mortality in ARDS, sepsis, and COVID-19–related critical illness, with several studies reporting independent associations with short-term mortality [19,20,21]. Similarly, CAR has been associated with disease severity and mortality in COVID-19 and general ICU populations, reflecting systemic inflammatory burden [22,23]. Together, these findings suggest that both LAR and CAR may provide valuable prognostic information in critically ill patients.

Despite growing interest in both indices, few studies have directly compared the prognostic performance of LAR and CAR within the same critically ill population, and data specifically focusing on COVID-19–associated ARDS remain limited. Moreover, the relative prognostic utility of biomarkers reflecting metabolic stress and tissue hypoxia compared with those reflecting systemic inflammatory burden remains unclear in this population, particularly during the early phase of critical illness. Comparative evaluation of these markers in this vulnerable population may provide clinically meaningful prognostic insight relevant to bedside clinical decision-making. The aim of the present study was to evaluate and compare the prognostic performance of LAR and CAR for 30-day mortality in critically ill patients with COVID-19–related ARDS. By focusing on biomarker measurements obtained within the first 48 h of ICU admission and evaluating their performance across ARDS severity strata, this study sought to assess their utility for early risk stratification. We further sought to evaluate their performance across clinically relevant subgroups and examine their overall prognostic value.

## 2. Materials and Methods

### 2.1. Study Design and Data Source

This was a retrospective cohort study using data from the Medical Information Mart for Intensive Care IV (MIMIC-IV, version 3.1) database [24,25]. The database contains de-identified clinical data from adult patients admitted to Beth Israel Deaconess Medical Center intensive care units. COVID-19 cases were identified using the international classification of diseases, tenth revision (ICD-10) diagnosis code (U07.1) from pandemic-era admissions represented in MIMIC-IV v3.1, which includes admissions occurring between 2020 and 2022.

### 2.2. Study Population, Cohort Selection and Variables

**Figure 1:**
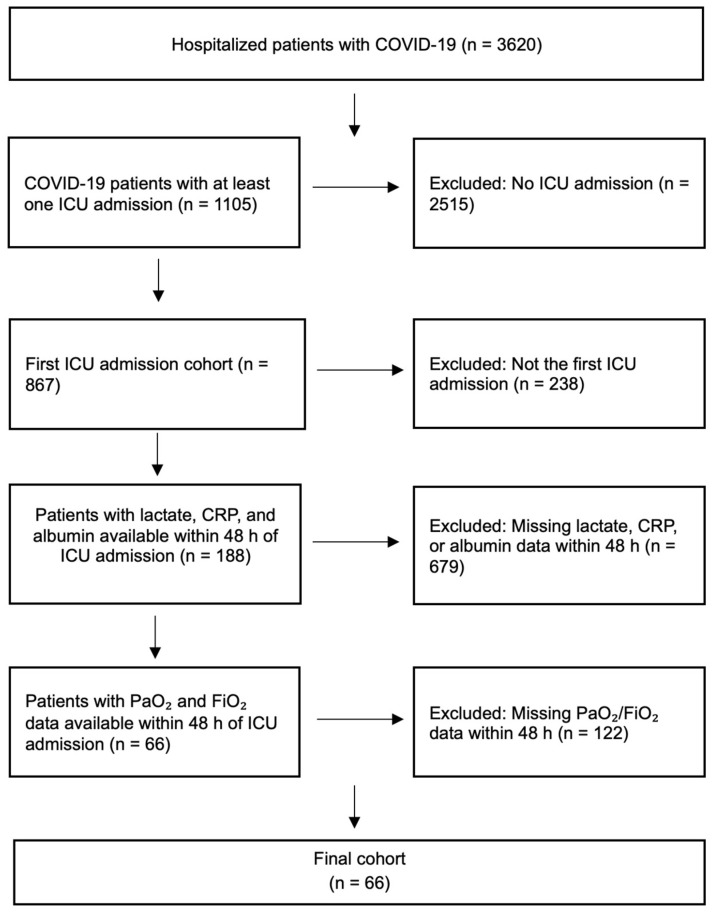
Flow diagram illustrating cohort selection from the MIMIC-IV v3.1 database. Only the first ICU admission per patient was included. Laboratory variables (lactate, C-reactive protein, and albumin) and arterial PaO_2_ and FiO_2_ measurements were obtained within the first 48 h of ICU admission and used to derive study variables.

Adult COVID-19 patients aged 18 years or older were eligible for inclusion. Among hospitalized COVID-19 patients, only those with at least one ICU admission were eligible for inclusion. Cohort selection was restricted to the first ICU admission for each patient to avoid duplicate observations. Patients were required to have serum lactate, C-reactive protein (CRP), and albumin measurements obtained within the first 48 h of ICU admission. Eligible patients were further required to have arterial PaO_2_ and corresponding FiO_2_ measurements within the same time window, from which the PaO_2_/FiO_2_ ratio was calculated. When multiple paired measurements were available within the first 48 h of ICU admission, the first available value was used for analysis. ARDS severity was classified according to the PaO_2_/FiO_2_ thresholds defined in the Berlin definition. Patients with missing lactate, CRP, albumin or PaO_2_/FiO_2_ ratio measurements within this time window were excluded from the analysis. No imputation of missing data was performed. The cohort selection process is shown in Figure 1. The following indices were calculated using early ICU laboratory values: CAR, defined as CRP divided by albumin, and LAR, defined as lactate divided by albumin. In the MIMIC-IV database, demographic characteristics, laboratory measurements, diagnoses, procedures, treatments, and outcome data are derived from the electronic health records of Beth Israel Deaconess Medical Center and are available as de-identified patient-level data. For the present study, relevant demographic, clinical, laboratory, oxygenation, and outcome variables were extracted from the database and used for cohort selection, calculation of study indices, and outcome assessment. The primary outcome was 30-day mortality following ICU admission. Mortality status was determined using the date of death recorded in the MIMIC-IV database. Mortality status and follow-up information were used to determine the primary outcome.

### 2.3. Statistical Analysis

Continuous variables are reported as mean ± standard deviation or median (interquartile range), and categorical variables as counts and percentages. Appropriate parametric or nonparametric tests were selected according to data distribution and variable characteristics for group comparisons. The prognostic performance of LAR and CAR for 30-day mortality was evaluated using receiver operating characteristic (ROC) curve analysis, with discrimination quantified by the area under the curve (AUC). Optimal cutoff values were determined using Youden’s index. Survival analyses were conducted using Kaplan–Meier curves and compared with the log-rank test. For survival analyses, time-to-event was defined as the interval from ICU admission to death within 30 days or censoring at 30 days. Cox proportional hazards regression models were used to estimate hazard ratios with corresponding confidence intervals, adjusting for clinically relevant covariates. Variables included in multivariable models were selected based on clinical relevance and prior literature. Given the limited sample size and number of outcome events, multivariable models were restricted to a small number of clinically relevant covariates to reduce the risk of model overfitting. However, the possibility of residual overfitting and instability of effect estimates cannot be excluded. A two-sided *p* value < 0.05 was considered statistically significant. Statistical analyses and figures were conducted using R software version 4.5.1 (R Foundation for Statistical Computing, Vienna, Austria).

## 3. Results

### 3.1. Baseline Characteristics

A total of 66 critically ill COVID-19 patients were included (Figure 1), of whom 36 survived and 30 died within 30 days of ICU admission. Non-survivors were significantly older than survivors (median age 67.0 vs. 56.0 years, *p* = 0.0487). Lactate levels were higher in non-survivors (1.9 vs. 1.6 mmol/L, *p* = 0.0264), while albumin levels were significantly lower (2.7 vs. 3.1 g/dL, *p* = 0.0301). Consequently, both CAR and LAR were significantly elevated among non-survivors compared with survivors (CAR: 72.33 vs. 46.58, *p* = 0.0303; LAR: 0.77 vs. 0.52, *p* = 0.00416). ICU length of stay was shorter in non-survivors than survivors (8.15 vs. 14.95 days, *p* = 0.00225). Cardiovascular disease was more prevalent among non-survivors than survivors (53.3% vs. 22.2%, *p* = 0.018). There were no significant differences between groups with respect to sex, hypertension, diabetes mellitus, obesity, chronic kidney disease, sepsis, vasoactive use, early mechanical ventilation, PaO_2_/FiO_2_ ratio, ARDS severity distribution or other baseline clinical parameters. Baseline characteristics are shown in Table 1.

### 3.2. Predictive Performance of LAR and CAR for 30-Day Mortality

**Figure 2:**
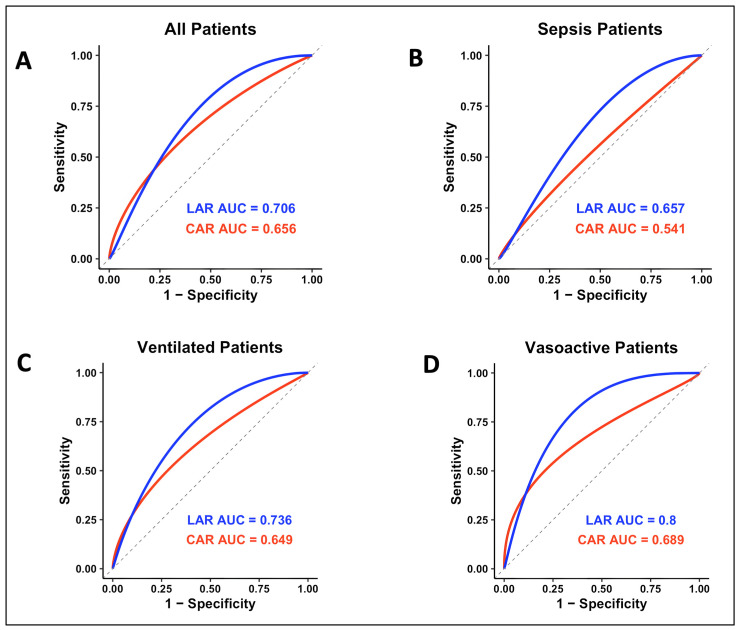
Receiver operating characteristic (ROC) curves for the lactate-to-albumin ratio (LAR) and the C-reactive protein-to-albumin ratio (CAR) for prediction of 30-day mortality in critically ill patients with COVID-19. (**A**) All patients (*n* = 66). (**B**) Sepsis patients (*n* = 40). (**C**) Ventilated patients within 48 h (*n* = 59). (**D**) Vasoactive use patients within 48 h (*n* = 39).

The discriminatory performance of LAR and CAR for predicting 30-day mortality is shown in Figure 2. In the overall cohort of COVID-19 patients, LAR demonstrated moderate predictive ability with an AUC of 0.706 (95% CI, 0.579–0.834), while CAR showed lower discrimination with an AUC of 0.656 (95% CI, 0.521–0.790) (Figure 2A). Among patients with sepsis, the predictive performance of both indices decreased; however, LAR showed higher discriminatory performance than CAR, with an AUC of 0.657 (95% CI, 0.482–0.831) compared with 0.541 (95% CI, 0.356–0.727) (Figure 2B). In patients requiring mechanical ventilation within the first 48 h of ICU admission, LAR again showed better discrimination (AUC 0.736; 95% CI, 0.605–0.866), whereas CAR demonstrated modest performance (AUC 0.649; 95% CI, 0.505–0.794) (Figure 2C). Among patients receiving vasoactive medications, both indices exhibited improved predictive ability, with LAR achieving an AUC of 0.80 (95% CI, 0.655–0.945) and CAR an AUC of 0.689 (95% CI, 0.519–0.860) (Figure 2D). In these subgroup analyses, LAR generally demonstrated higher discriminatory performance than CAR.

### 3.3. Predictive Performance of LAR and CAR Stratified by Age and Sex

**Figure 3:**
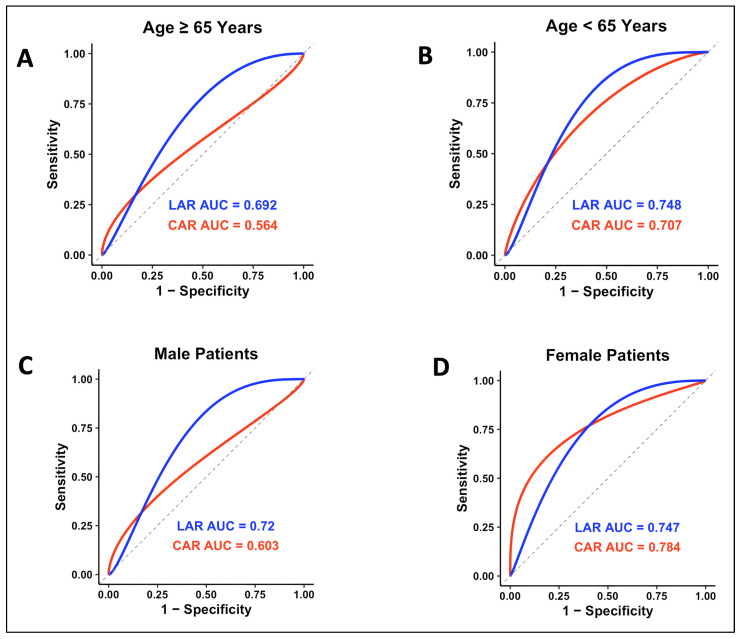
Receiver operating characteristic (ROC) curves for LAR and CAR in age- and sex-based subgroups. (**A**) Patients aged 65 years or older (*n* = 33). (**B**) Patients younger than 65 years (*n* = 33). (**C**) Male patients (*n* = 39). (**D**) Female patients (*n* = 27).

Age- and sex-stratified analyses showed differential predictive performance for LAR and CAR (Figure 3). In patients aged 65 years or more, LAR demonstrated moderate discrimination (AUC 0.692; 95% CI, 0.495–0.889), whereas CAR showed limited performance (AUC 0.564; 95% CI, 0.363–0.765) (Figure 3A). In patients younger than 65 years, both indices performed better, with LAR achieving an AUC of 0.748 (95% CI, 0.566–0.930) and CAR an AUC of 0.707 (95% CI, 0.512–0.901) (Figure 3B). Among male patients, LAR performed better than CAR (AUC 0.720; 95% CI, 0.553–0.887) vs. (AUC 0.603; 95% CI, 0.404–0.802) (Figure 3C), whereas in female patients, CAR showed slightly higher discrimination (AUC 0.784; 95% CI, 0.605–0.963) compared with LAR (AUC 0.747; 95% CI, 0.546–0.949) (Figure 3D).

### 3.4. Predictive Performance of LAR and CAR Stratified by ARDS

**Figure 4:**
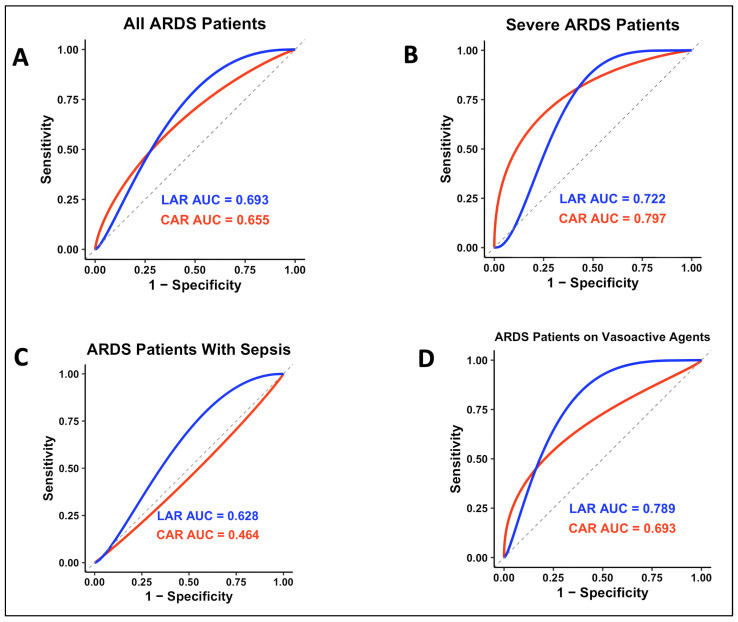
Receiver operating characteristic (ROC) curves for LAR and CAR in ARDS-related subgroups. (**A**) All ARDS patients (*n* = 63). (**B**) Severe ARDS patients (*n* = 40). (**C**) ARDS patients with sepsis (*n* = 38). (**D**) ARDS patients receiving vasoactive agents (*n* = 36).

Among patients with ARDS, LAR demonstrated moderate discriminatory ability for predicting 30-day mortality with an AUC of 0.693 (95% CI, 0.560–0.825), while CAR showed slightly lower performance with an AUC of 0.655 (95% CI, 0.518–0.793) (Figure 4A). In the subgroup of patients with severe ARDS, both indices performed better, with CAR demonstrating higher discrimination (AUC 0.797; 95% CI, 0.655–0.939) compared with LAR (AUC 0.722; 95% CI, 0.549–0.895) (Figure 4B). Among ARDS patients with concomitant sepsis, the predictive performance of both markers decreased; however, LAR maintained modest discrimination (AUC 0.628; 95% CI, 0.445–0.811), whereas CAR demonstrated limited discriminatory ability (AUC 0.464; 95% CI, 0.274–0.654) (Figure 4C). In ARDS patients receiving vasoactive agents, both indices again showed improved performance, with LAR achieving an AUC of 0.789 (95% CI, 0.631–0.948) and CAR an AUC of 0.693 (95% CI, 0.517–0.870) (Figure 4D). Given that both LAR and CAR demonstrated better discriminatory performance in patients with severe ARDS, optimal cutoff values were derived from this subgroup. The Youden index identified an optimal cutoff of 0.53 for LAR (sensitivity 85.7%, specificity 63.2%) and 45.9 for CAR (sensitivity 90.5%, specificity 63.2). These thresholds were subsequently used to stratify patients in the survival analyses.

### 3.5. Survival Analysis Based on LAR and CAR Cutoff Values

**Figure 5:**
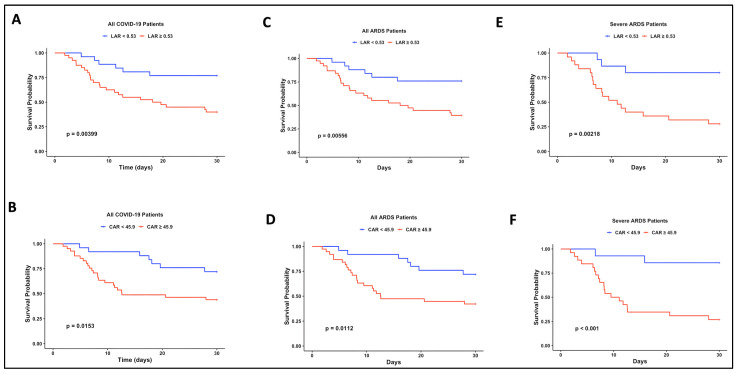
Kaplan–Meier survival curves stratified by LAR and CAR cutoff values. (**A**) All COVID-19 patients stratified by LAR level. Low LAR (*n* = 26) and high LAR (*n* = 40). (**B**) All COVID-19 patients stratified by CAR level. Low CAR (*n* = 25) and high CAR (*n* = 41). (**C**) All ARDS patients stratified by LAR level. Low LAR (*n* = 25) and high LAR (*n* = 38). (**D**) All ARDS patients stratified by CAR level. Low CAR (*n* = 25) and high CAR (*n* = 38). (**E**) Severe ARDS patients stratified by LAR level. Low LAR (*n* = 15) and high LAR (*n* = 25). (**F**) Severe ARDS patients stratified by CAR level. Low CAR (*n* = 14) and high CAR (*n* = 26).

Kaplan–Meier analyses demonstrated significant differences in 30-day mortality when patients were stratified using the best cutoff values (Figure 5). Among all COVID-19 patients, those with high LAR had significantly higher 30-day mortality compared with those with low LAR (60.0% vs. 23.1%; *p* = 0.004) (Figure 5A). Similarly, patients with high CAR exhibited higher mortality than those with low CAR (56.1% vs. 28.0%; *p* = 0.0153) (Figure 5B).

Among all ARDS patients, high LAR was associated with a markedly increased 30-day mortality compared with low LAR (60.5% vs. 24.0%; *p* = 0.00556) (Figure 5C). A comparable pattern was observed for CAR, with higher mortality in patients with high CAR than in those with lower values (57.9% vs. 28.0%; *p* = 0.0112) (Figure 5D).

In patients with severe ARDS, mortality differences were even more pronounced. Patients with high LAR had substantially higher 30-day mortality compared with those with low LAR (72.0% vs. 20.0%; *p* = 0.00218) (Figure 5E). Likewise, patients with high CAR experienced significantly higher mortality than those with low CAR levels (73.1% vs. 14.3%; *p* < 0.001) (Figure 5F).

### 3.6. Independent Predictors of 30-Day Mortality

To evaluate factors associated with 30-day mortality, multivariable Cox proportional hazards models were constructed (Table 2). LAR remained independently associated with increased 30-day mortality across all models, including the overall cohort (HR 1.85; 95% CI, 1.33–2.60; *p* < 0.001), all ARDS patients (HR 1.88; 95% CI, 1.27–2.80; *p* = 0.001), and patients with severe ARDS (HR 1.78; 95% CI, 1.15–2.70; *p* = 0.009). In contrast, CAR was not independently associated with mortality after multivariable adjustment. Female sex was independently associated with higher mortality in the overall COVID-19 and ARDS cohorts but not in severe ARDS. Age, PaO_2_/FiO_2_ ratio, ventilation status, vasoactive use, and sepsis were not independently predictive of 30-day mortality.

## 4. Discussion

In this retrospective cohort of critically ill patients with COVID-19, we evaluated the prognostic performance of LAR and CAR for predicting 30-day mortality, with particular attention to ARDS severity. Overall, LAR demonstrated higher discriminatory performance than CAR in the overall cohort and most clinically relevant subgroups. Although both markers showed their better discrimination among patients with severe ARDS, LAR maintained similar performance across several clinical subgroups. Cutoff values derived from the severe ARDS subgroup were associated with differences in 30-day mortality in subsequent survival analyses. In multivariable models, LAR remained independently associated with 30-day mortality across all cohorts, whereas CAR did not retain independent prognostic significance. Taken together, these findings suggest that LAR may provide additional prognostic value in critically ill COVID-19 patients and could serve as a useful tool for early stratification in those patients with COVID-19 associated with ARDS.

Previous studies have demonstrated the prognostic value of LAR across critical illness, including ARDS and COVID-19–related respiratory failure. In a large ARDS cohort, Wang et al. showed that LAR was independently associated with 28-day mortality and provided better discrimination than lactate or albumin alone, with performance comparable to established severity scores [21]. In critically ill patients with COVID-19, Kokkoris et al. similarly showed that LAR was independently associated with ICU mortality and outperformed lactate alone in ROC analyses across key clinical subgroups [20]. Additional ICU studies involving patients with acute respiratory failure and sepsis have further supported LAR as a prognostic marker for mortality, mechanical ventilation, and vasopressor requirements, highlighting its ability to reflect both metabolic stress and disease progression [26]. In line with this literature, our findings suggest that LAR may provide clinically relevant prognostic information in critically ill COVID-19 patients. Notably, its discriminatory performance was strongest among patients with ARDS, supporting its potential role as a risk stratification tool in this high-risk population.

Compared with LAR, the CRP-to-albumin ratio has been investigated less extensively in ARDS and COVID-19–related critical illness, with most available evidence derived from heterogeneous or non-ARDS cohorts. Early COVID-19 studies demonstrated that CAR was associated with disease severity, intensive care requirement, and mortality, with higher CAR values observed among patients with worse clinical outcomes [23]. In smaller hospital-based cohorts, CAR showed strong diagnostic performance for identifying severe COVID-19, with high sensitivity and specificity when used as a marker of systemic inflammation and disease severity [27]. Beyond COVID-19, CAR has been evaluated in general ICU populations, where it was independently associated with 28-day mortality and provided modest improvement over CRP alone, supporting its role as a prognostic indicator [22]. However, many CAR-focused studies have emphasized early severity classification or mixed ICU populations, with limited stratification by ARDS severity. In this context, our findings add to the existing literature by evaluating CAR specifically in COVID-19–associated ARDS. Although CAR demonstrated moderate discriminatory ability among patients with severe ARDS, its prognostic performance was less consistent across clinical subgroups and did not remain independently associated with 30-day mortality after multivariable adjustment. These findings suggest that, while CAR may reflect inflammatory burden and disease severity, its prognostic utility appears to be more limited than that of LAR in this patient population.

Several studies have directly evaluated LAR and CAR within the same cohorts, allowing comparative assessment of their prognostic performance. In a large multicenter emergency department cohort of patients with sepsis and septic shock, Yoo et al. demonstrated that LAR outperformed CAR and quick sequential organ failure assessment (qSOFA) in predicting 28-day mortality in both univariate and multivariate analyses [28]. Similarly, in a prospective study of infected patients presenting to the emergency department, Turcato et al. reported that both LAR and CAR were independently associated with 30-day mortality, although albumin alone showed the highest discrimination, followed by LAR and then CAR [29]. These studies suggest that while both ratios capture complementary aspects of systemic inflammation and metabolic stress, LAR has generally demonstrated more favorable prognostic performance when directly compared with CAR. Consistent with these observations, our study found that LAR had higher discrimination ability than CAR in most subgroups and remained independently associated with mortality after multivariable adjustment. Furthermore, our findings extend previous work by comparing both biomarkers within a relatively homogeneous cohort of critically ill patients with COVID-19. While CAR retained prognostic relevance in certain subgroups, LAR showed more consistent performance across clinical strata and remained independently associated with 30-day mortality after multivariable adjustment.

The differential performance of LAR and CAR likely reflects the distinct biological processes captured by each marker. LAR integrates serum lactate, a surrogate of tissue hypoxia and impaired perfusion, with albumin, which reflects both nutritional status and systemic inflammation [30]. As such, LAR may more directly represent acute metabolic stress and circulatory dysfunction, which are central features of severe ARDS and critical COVID-19 [31,32]. In contrast, CAR primarily reflects systemic inflammatory burden and hepatic protein synthesis, which may be influenced by timing of measurement, pre-existing conditions, and variability in inflammatory responses [33,34]. These biological differences may partially explain the observed differences in prognostic performance in our cohort. More broadly, adverse outcomes in COVID-19 are influenced not only by the acute inflammatory response but also by underlying comorbidities, cardiovascular vulnerability, physiological reserve and overall illness burden [35,36]. These considerations suggest that the prognostic value of such biomarkers may reflect broader cardiometabolic, inflammatory, and physiological vulnerability rather than a COVID-19–specific mortality signal.

ARDS is classified into mild, moderate, and severe forms based on the PaO_2_/FiO_2_ ratio, with severe ARDS representing the most critical stage of lung injury, characterized by profound hypoxemia, extensive lung damage, and high mortality [37]. In COVID-19–induced ARDS, particularly in severe cases, these clinical manifestations are often more pronounced, reflecting greater hypoxemia, inflammatory burden, and organ stress, thereby rendering this population highly vulnerable to adverse outcomes [38]. In our COVID-19 cohort, both LAR and CAR showed better discriminatory performance in the severe ARDS subgroup. Consequently, cutoff values were derived from this subgroup and applied in subsequent survival analyses. LAR remained independently associated with 30-day mortality after multivariable adjustment.

In our cohort, CAR showed relatively better discrimination in female patients. Although this finding should be interpreted cautiously given the limited sample size of the subgroup, several biological mechanisms may contribute to the observed difference. Females tend to mount a stronger CRP response to inflammatory stimuli than males, influenced by sex hormones and immune regulatory differences, which may enhance the prognostic value of CRP-based markers [39,40,41]. In addition, albumin levels appeared to be lower in women than men during critical illness, which can further amplify CAR values [42]. Further studies are needed to determine whether the observed sex-specific differences are reproducible in larger cohorts.

Most studies of COVID-19 have reported higher disease severity and mortality among males compared with females. Early clinical reports and population-based data consistently showed that men experienced worse outcomes and higher mortality than women, independent of age and comorbidities [43]. Subsequent reviews have supported this male predominance, suggesting contributions from differences in immune responses, viral clearance, and hormonal factors, although these mechanisms remain incompletely understood [44]. However, these findings largely reflect heterogeneous COVID-19 populations and are not specific to patients with established ARDS. Evidence from ARDS-focused cohorts indicates that sex-related differences in mortality may change with disease severity. In the LUNG SAFE prospective study, overall mortality was similar between sexes, but female sex was independently associated with higher mortality in severe ARDS [45]. Given that our cohort was enriched for ARDS and focused on patients with available PaO_2_/FiO_2_ measurements, these observations may provide context for the association between female sex and mortality observed in our analysis.

In our COVID-19 cohort, non-survivors had a significantly shorter ICU length of stay than survivors, which may reflect early mortality rather than faster clinical recovery. Similar findings have been reported by Lavrentieva et al., who conducted a large prospective observational study of mechanically ventilated COVID-19 patients and observed shorter ICU stays among non-survivors [46]. Likewise, a recent systematic review and meta-analysis by Zhang et al. evaluating outcomes in patients with severe ARDS, including COVID-19–related ARDS receiving extracorporeal membrane oxygenation (ECMO), demonstrated that non-survivors had significantly shorter hospital and ICU lengths of stay compared with survivors, supporting the interpretation that shorter ICU stays likely reflect early death rather than clinical improvement [47].

This study has several strengths and limitations. A key strength is the use of the MIMIC-IV database, which provides granular, standardized clinical and laboratory data for critically ill patients. Biomarker measurements were restricted to values obtained within the first 48 h of ICU admission, ensuring temporal consistency and clinical relevance. Requiring the availability of lactate, albumin, CRP, and PaO_2_/FiO_2_ measurements within this early window allowed uniform ARDS classification and biologically meaningful evaluation of prognostic markers, albeit at the expense of a reduced sample size. In addition, LAR and CAR were evaluated across clinically relevant subgroups using multivariable models. However, the retrospective design limits causal inference and may be subject to residual confounding. The strict inclusion criteria, including the requirement for lactate, CRP, albumin, and PaO_2_/FiO_2_ measurements within the predefined study window, may have substantially reduced the eligible study population, potentially introducing selection bias. The relatively small sample size and limited number of outcome events may have reduced statistical power and contributed to instability of effect estimates, particularly in subgroup and multivariable analyses. A further limitation is the absence of established severity-of-illness scores such as qSOFA, acute physiology and chronic health evaluation (APACHE), and simplified acute physiology score II (SAPS II). These scores were not available as pre-calculated variables in the study dataset, and calculation of such scores requires multiple physiological and laboratory variables that were not consistently available within the predefined study window. Additionally, given the limited sample size and number of outcome events, inclusion of additional severity indices could have increased the risk of model overfitting. Nevertheless, the possibility of residual overfitting cannot be excluded. External validation in independent cohorts is also required to confirm the generalizability of these findings. Because the cutoff values were derived and evaluated within the same dataset, the reported thresholds should be considered exploratory rather than definitive clinical cutoffs. In addition, COVID-19 management evolved substantially during the pandemic, and temporal differences in treatment practices may have influenced patients’ outcomes. Lastly, ARDS severity was classified retrospectively using PaO_2_/FiO_2_ thresholds, and complete verification of all Berlin definition criteria was not possible within the study design.

## 5. Conclusions

Among critically ill patients with COVID-19, LAR demonstrated independent prognostic value for 30-day mortality across multiple clinical settings, whereas CAR exhibited more context-dependent prognostic performance. Severe ARDS emerged as the most informative subgroup for risk stratification, and cutoff values derived from this population were associated with significant differences in mortality risk in subsequent survival analyses. Collectively, these findings suggest that readily available biomarkers, particularly LAR, may provide useful prognostic information and support early risk stratification in patients with COVID-19–associated ARDS.

The superior and more consistent performance of LAR may reflect its ability to integrate information from two complementary pathophysiological domains: tissue hypoperfusion and metabolic stress, represented by lactate, and nutritional and inflammatory status, reflected by serum albumin. As both laboratory parameters are routinely measured in critically ill patients, LAR can be calculated easily without additional cost or testing, making it a potentially practical tool for bedside risk assessment. Furthermore, the ability of LAR to retain prognostic significance after adjustment for established clinical variables suggests that it may provide information beyond conventional markers of disease severity.

Nevertheless, external validation in larger and more diverse cohorts is required before routine clinical implementation can be recommended. Future prospective studies should investigate whether integrating LAR with established severity scores and other clinical variables improves prognostic accuracy and risk stratification in patients with COVID-19–associated ARDS. In addition, studies evaluating serial measurements of LAR may help determine whether dynamic changes over time provide incremental prognostic value and could be used to monitor disease progression or response to treatment.

## Figures and Tables

**Table 1:** Baseline demographic, clinical, and laboratory characteristics of COVID-19 patients.

Variable	All Patients (*n* = 66)	Survivors (*n* = 36)	Non-Survivors (*n* = 30)	*p*-Value
Age (years)	64.5 [49.25–73.75]	56.0 [38.75–74.0]	67.0 [56.5–72.75]	0.0487
Male *n* (%)	39 (59.1%)	25 (69.4%)	14 (46.7%)	0.105
Female *n* (%)	27 (40.9%)	11 (30.6%)	16 (53.3%)	
Hypertension, *n* (%)	33 (50.0)	16 (44.4)	17 (56.7)	0.458
Diabetes mellitus, *n* (%)	30 (45.5)	14 (38.9)	16 (53.3)	0.355
Obesity, *n* (%)	26 (39.4)	16 (44.4)	10 (33.3)	0.505
Chronic kidney disease, *n* (%)	15 (22.7)	5 (13.9)	10 (33.3)	0.114
Cardiovascular disease, *n* (%)	24 (36.4)	8 (22.2)	16 (53.3)	0.018
Sepsis *n* (%)	40 (60.6%)	21 (58.3%)	19 (63.3%)	0.872
Vasoactive use within 48	39 (59.1%)	19 (52.8%)	20 (66.7%)	0.373
Ventilated within 48 h	59 (89.4%)	32 (88.9%)	27 (90.0%)	1.000
PaO_2_/FiO_2_ ratio	89.05 [66.38–135.98]	96.61 [69.5–157.5]	84.11 [61.69–110.42]	0.239
ARDS severity				
Mild	8 (12.1%)	4 (11.1%)	4 (13.3%)	
Moderate	15 (22.7%)	11 (30.6%)	4 (13.3%)	
Severe	40 (60.6%)	19 (52.8%)	21 (70.0%)	
CRP (mg/L)	167.7 [103.05–230.63]	146.3 [85.03–209.7]	200.45 [132.62–233.47]	0.115
Lactate (mmol/L)	1.7 [1.42–2.58]	1.6 [1.28–1.9]	1.9 [1.52–3.0]	0.0264
Albumin (g/dL)	2.9 [2.5–3.38]	3.1 [2.8–3.4]	2.7 [2.3–3.0]	0.0301
CAR	61.72 [31.94–83.52]	46.58 [29.86–75.57]	72.33 [46.98–101.21]	0.0303
LAR	0.55 [0.49–0.97]	0.52 [0.43–0.65]	0.77 [0.54–1.09]	0.00416
Ventilation days	9.0 [5.0–16.75]	12.0 [6.0–19.25]	7.0 [4.0–11.75]	0.0699
ICU LOS (days)	11.94 [7.07–19.7]	14.95 [10.71–23.24]	8.15 [4.02–12.72]	0.00225

Continuous variables are presented as median [interquartile range], and categorical variables as number (percentage). “Within 48 h” refer to within the first 48 h of ICU admission. Abbreviations: PaO_2_, arterial partial pressure of oxygen; FiO_2_, fraction of inspired oxygen; ARDS, acute respiratory distress syndrome; CRP, C-reactive protein; CAR, C-reactive protein-to-albumin ratio; LAR, lactate-to-albumin ratio; LOS, length of stay; ICU, intensive care unit.

**Table 2:** Multivariable Cox proportional hazards models for 30-day mortality in COVID-19 patients, ARDS patients, and severe ARDS patients.

All COVID-19 Patients (*n* = 66)			
Variable	Hazard Ratio (HR)	95% Confidence Interval	*p* Value
LAR	1.85	1.33–2.60	<0.001
CAR	1.01	1.00–1.01	0.200
Age (years)	1.02	1.00–1.10	0.061
PaO_2_/FiO_2_ ratio	1.00	0.99–1.00	0.240
Female (vs. male)	2.33	1.10–4.90	0.026
Ventilation status	1.20	0.30–4.90	0.797
Vasoactive use	0.93	0.34–2.50	0.887
Sepsis	0.64	0.27–1.50	0.317
All ARDS Patients (*n* = 63)			
LAR	1.88	1.27–2.80	0.001
CAR	1.01	1.00–1.01	0.185
Age (years)	1.02	1.00–1.10	0.067
PaO_2_/FiO_2_ ratio	1.00	0.99–1.00	0.739
Female (vs. male)	2.32	1.07–5.00	0.033
Ventilation status	1.09	0.26–4.60	0.904
Vasoactive use	1.00	0.36–2.80	0.994
Sepsis	0.63	0.26–1.50	0.311
Severe ARDS Patients (*n* = 40)			
LAR	1.78	1.15–2.70	0.009
CAR	1.01	1.00–1.01	0.055
Age (years)	1.01	0.98–1.10	0.411
PaO_2_/FiO_2_ ratio	0.99	0.96–1.00	0.336
Female (vs. male)	1.69	0.68–4.20	0.258
Ventilation status	1.11	0.18–6.90	0.908
Vasoactive use	1.52	0.36–6.30	0.568
Sepsis	0.67	0.23–2.00	0.466

Multivariable Cox proportional hazards models evaluating independent predictors of 30-day mortality in all COVID-19 patients (*n* = 66), all ARDS patients (*n* = 63), and severe ARDS patients (*n* = 40). Hazard ratios (HRs) with 95% confidence intervals are reported for LAR, CAR, age, PaO_2_/FiO_2_ ratio, sex, ventilation status, vasoactive use, and sepsis.

## Data Availability

The data used in this study are available from the MIMIC-IV v3.1 database, which is publicly accessible to qualified researchers through PhysioNet after completing the required training and data use agreement. The filtered datasets generated and analyzed during this study are available from the corresponding author upon reasonable request.
